# Bayesian inference for the onset time and epidemiological characteristics of emerging infectious diseases

**DOI:** 10.3389/fpubh.2024.1406566

**Published:** 2024-05-17

**Authors:** Benyun Shi, Sanguo Yang, Qi Tan, Lian Zhou, Yang Liu, Xiaohong Zhou, Jiming Liu

**Affiliations:** ^1^College of Computer and Information Engineering, Nanjing Tech University, Nanjing, China; ^2^College of Artificial Intelligence, Nanjing Tech University, Nanjing, China; ^3^Department of Computer Science, Hong Kong Baptist University, Hong Kong, China; ^4^Center for Disease Control and Prevention of Jiangsu Province, Nanjing, China; ^5^Department of Pathogen Biology, School of Public Health, Southern Medical University, Guangzhou, China

**Keywords:** Bayesian inference, emerging infectious disease (EID), epidemiological characteristics, Particle Markov chain Monte Carlo, SEIR compartmental model

## Abstract

**Background:**

Emerging infectious diseases pose a significant threat to global public health. Timely detection and response are crucial in mitigating the spread of such epidemics. Inferring the onset time and epidemiological characteristics is vital for accelerating early interventions, but accurately predicting these parameters in the early stages remains challenging.

**Methods:**

We introduce a Bayesian inference method to fit epidemic models to time series data based on state-space modeling, employing a stochastic Susceptible-Exposed-Infectious-Removed (SEIR) model for transmission dynamics analysis. Our approach uses the particle Markov chain Monte Carlo (PMCMC) method to estimate key epidemiological parameters, including the onset time, the transmission rate, and the recovery rate. The PMCMC algorithm integrates the advantageous aspects of both MCMC and particle filtering methodologies to yield a computationally feasible and effective means of approximating the likelihood function, especially when it is computationally intractable.

**Results:**

To validate the proposed method, we conduct case studies on COVID-19 outbreaks in Wuhan, Shanghai and Nanjing, China, respectively. Using early-stage case reports, the PMCMC algorithm accurately predicted the onset time, key epidemiological parameters, and the basic reproduction number. These findings are consistent with empirical studies and the literature.

**Conclusion:**

This study presents a robust Bayesian inference method for the timely investigation of emerging infectious diseases. By accurately estimating the onset time and essential epidemiological parameters, our approach is versatile and efficient, extending its utility beyond COVID-19.

## Background

The spread of infectious diseases has always been a major concern for public health and economic stability. Identifying epidemiological characteristics such as the onset time (i.e., the date of appearance of patient zero), the basic reproduction number, the latent period, and the infectious period in the early stages of an epidemic is essential for effective disease intervention and control. However, accurately predicting these factors is a difficult task (1, 2). The complexity of epidemiological dynamics is largely due to its stochastic nature, which is affected by various factors such as demographic characteristics (3), environmental conditions (4, 5), and human behaviors (6–8). Furthermore, indirect and subjective elements further complicate this process. For example, measurement errors, often caused by human activities, can lead to under-reporting or inaccuracies in the data. This problem is especially pronounced in the early stages of an epidemic, when surveillance methods and case reporting systems are still being developed and improved (9).

In epidemiological research, compartmental models have been widely used to simulate the transmission of infectious diseases, organizing the population into categories such as susceptible, infectious, and recovered (10). One significant benefit of these models is their ability to provide preliminary insights into the pace of disease spread, even when observational data is scarce in the initial phases of an epidemic (11, 12). Although beneficial, the deterministic nature of traditional compartmental models often oversimplifies the complexity of the dynamics of real-world diseases. To better capture the intricacies of epidemic spread, such as randomness and non-linear interactions, researchers have developed a range of stochastic compartmental models (13, 14). The early stages of an outbreak, marked by uncertain transmission mechanisms and flawed monitoring systems that produce incomplete data, emphasize the importance of stochastic models to more effectively simulate the uncertainties and variability of epidemic spread (15).

To estimate the onset time and explore epidemiological characteristics in the early stages of emerging infectious diseases, we simulate the dynamics of disease transmission and the observation of infected cases on the basis of state-space modeling. Specifically, a stochastic Susceptible-Exposed-Infectious-Removed (SEIR) model is used as the disease transmission process model, while a probabilistic distribution is used as the case detection observation model. In doing so, epidemiological parameters, as well as the onset time of the outbreak, can be inferred by fitting the state-space model to the time series of observed cases using a Bayesian inference approach. The integration of real-time data adjustments and machine learning algorithms could improve the accuracy of predictions, even with limited and imperfect data available in the early stages of an epidemic (16, 17). Specifically, in this paper, we adopt the particle Markov chain Monte Carlo (PMCMC) method to infer epidemiological parameters in the state-space model (18).

The PMCMC leverages the strength of Markov chain Monte Carlo (MCMC) in efficiently exploring high-dimensional parameter spaces and the ability of particle filters to handle the underlying dynamics of disease transmission, making it possible to accurately estimate parameters in intricate, non-linear epidemic models with latent variables or unobserved states. The MCMC component constructs a Markov chain that has the target posterior distribution of the model parameters as its equilibrium distribution. Through iterative sampling, MCMC explores the parameter space, generating a sequence of parameter values. Meanwhile, the particle filter component is used to simulate a set of potential solutions (particles) that evolve over time, considering the observed data. Each particle represents a possible state of the system being modeled, and its weight is adjusted on the basis of how well it fits the observed data. The particle filter is used within each iteration of the MCMC algorithm to provide an estimate of the likelihood for the current set of parameters. This likelihood estimation is crucial for the MCMC to decide whether to accept or reject the new parameter values. The synergy between MCMC and particle filters in PMCMC leverages the strength of MCMC to efficiently explore high-dimensional parameter spaces and the ability of particle filters to handle the underlying dynamics of disease transmission with latent variables or unobserved states, especially for newly emerging infectious diseases.

In this study, we first validate the efficacy of the PMCMC algorithm in determining the epidemiological parameters of infectious diseases through synthetic experiments. Then we apply the proposed model and algorithm to real-world case studies, that is, to conduct retrospective investigations on COVID-19 outbreaks in Wuhan, Shanghai and Nanjing, China, respectively. We estimate key epidemiological parameters for each outbreak, including the basic reproduction number, the latent period, the infectious period, and the onset time. We corroborate the accuracy of our results by comparing them with existing research and survey data. This comparison not only underscores the effectiveness of our approach, but also highlights the potential of our model in addressing future emerging infectious disease outbreaks.

## Methods

###  State-space modeling of epidemiological dynamics

Because epidemiological dynamics are inherently stochastic and can be influenced by various factors (e.g., genetics, environmental factors, and social behavior), we introduce noise associated with variability in the transmission rate in a Susceptible-Exposed-Infectious-Removed (SEIR) model to simulate epidemic dynamics forward in time:


(1) $$\begin{array}{l} S_{t+1}=S_{t}-\left(1+F\xi \right)\beta \frac{S_{t}}{N}I_{t}\\ E_{t+1}=E_{t}+\left(1+F\xi \right)\beta \frac{S_{t}}{N}I_{t}-\alpha E_{t}\\ I_{t+1}=I_{t}+\alpha E_{t}-\gamma I_{t}\\ R_{t+1}=R_{t}+\gamma I_{t} \end{array}$$

where *S*_*t*_, *E*_*t*_, *I*_*t*_, and *R*_*t*_ represent the number of susceptible, exposed, infectious, and removed individuals at the time *t*, *N* is the population size, the noise term ξ is a normal random variable with mean equals zero and variance equals one, and *F* is a constant noise magnitude. Because the main focus of our study is on the early stages of an epidemic, we assume that the total population size is constant, that is, *N* = *S*_*t*_+*E*_*t*_+*I*_*t*_+*R*_*t*_ at any time *t*. Furthermore, the transmission coefficient β measures the probability that an infectious person will transmit a disease to a susceptible person during a single contact, multiplied by the average number of contacts per person per unit of time. The symbol α = 1/*D*_*E*_ is the rate at which individuals move from the latent stage to the infectious stage, where *D*_*E*_ represents the mean latent period, that is, the average time between infection and the onset of infectiousness. The symbol γ = 1/*D*_*I*_ represents the recovery rate, where *D*_*I*_ is the mean infectious period [i.e., the serial interval minus the mean latent period (19)]. Consequently, the basic reproduction number *R*_0_ of the SEIR model can be determined as *R*_0_ = β/γ. Since we focus solely on the epidemiological characteristics of infectious diseases in their early stages, it is reasonable to assume that the parameters mentioned above (i.e., β, *D*_*E*_, and *D*_*I*_) remain constant during this period.

In this paper, we use a general statistical framework to infer epidemiological parameters, especially the onset time of an emerging epidemic, by fitting stochastic epidemic models to the time series of case report data based on state-space modeling. State-space models (SSMs) consist of a process model and an observation model. The process model describes the underlying dynamics of the state variables *x*_*t*_ (that is, unknown latent variables) as a Markov process with a set of model parameters θ for all time points *t* in {1, ⋯ , *T*}: *x*_*t*_~*p*(*x*_*t*_|*x*_*t*−1_, θ). Specifically, we use the SEIR model mentioned above (i.e., Equation 1) as the process model for the underlying transmission dynamics of an infectious disease with state variables *x*_*t*_ = {*S*_*t*_, *E*_*t*_, *I*_*t*_, *R*_*t*_}. By setting the onset time of an epidemic (i.e., *d* days before the first reported case), we can simulate the state variables *x*_*t*_ and track the cumulative number of infected individuals over time, i.e., *C*_*t*+1_ = *C*_*t*_+(1+*Fξ*)β*S*_*t*_*I*_*t*_/*N*. In other words, the number of new infections is Δ*c*_*t*_ = (1+*Fξ*)β*S*_*t*_*I*_*t*_/*N*.

On the other hand, the observation model in SSMs relates the observed data (time series of reported cases) to the underlying process model. If we assume that only a proportion ρ of newly infected cases is detected and the observation error follows a normal distribution, then the probability of observing *z*_*t*_ cases at a specific time *t* can be expressed using the following observation model (16):


$$\begin{array}{l} p\left(z_{t}|\Delta c_{t}\right)=N\left(z_{t}|\rho \Delta c_{t},\tau \rho \Delta c_{t}\right), \end{array}$$

where N(·) is a normal distribution with mean μ = ρ*Δc*_*t*_ and observation variance $\sigma^{2}=\tau \rho \Delta c_{t}$, and a scaling parameter τ. In this case, the model parameters θ contain all the parameters of the SEIR model (i.e., α, β, γ, and *d*) as well as the observation model parameters ρ and τ. Table 1 provides a summary of all parameters and their descriptions used in the state-space model. In summary, the observation model can be described as follows:


$$\begin{array}{l} z_{t}\sim p\left(z_{t}|x_{t},\theta \right). \end{array}$$

It is important to note that our assumptions rely on the reporting of confirmed cases with the same day, with no delays exceeding one day. Examining the impact of delayed case reporting on the inference of epidemiological parameters is a more intricate challenge that we plan to tackle in future studies.

**Table 1:** Parameters and descriptions of the proposed state-space model.

**Parameters**	**Descriptions**	**Values**
α	The transition rate from latent stage to infectious stage	α = 1/*D*_*E*_
*D*_*E*_	The mean latent period	To be estimated
γ	The recovery rate	γ = 1/*D*_*I*_
*D*_*I*_	The mean infectious period	To be estimated
β	The transmission rate	β = *R*_0_/γ
*R*_0_	The basic reproduction number	To be estimated
*d*	The number of days before the first confirmed infection	To be estimated
ρ	A parameter in the observation model	To be estimated
τ	A scaling parameter in the obervation model	To be estimated
θ	The set of model parameters	θ = {*R*_0_, *D*_*E*_, *D*_*I*_, *d*, ρ, τ}
*x*_*t*_	The simulation output of the SEIR model at time *t*	Hidden^a^
*z*_*t*_	The number of observed new infections at time *t*	Observed cases^b^
*N*	The population size	NBS^c^

^a^The simulated time series based on the SEIR model (i.e., Equation 1) with given parameters in θ. ^b^The time series of reported cases of COVID-19 in Wuhan were obtained from (20). The time series of reported COVID-19 cases in Shanghai and Nanjing were obtained from National Health Commission of the People's Republic of China (NHC: http://www.nhc.gov.cn/xcs/xxgzbd/gzbd_index.shtml) and Jiangsu Commission of Health (JSCH: http://wjw.jiangsu.gov.cn/) ^c^The population size in this study is obtained from the National Bureau of Statistics (NBS).

### Bayesian inference with Markov chain Monte Carlo method

To fit the state-space model to time series of observed data *z*_1:*T*_, we use a Bayesian inference approach. Given the time series of reported cases *z*_1:*T*_, the posterior density of the parameter θ and the latent states *x*_1:*T*_ can be computed as follows:


(2) $$\begin{array}{l} p\left(\theta ,x_{1:T}|z_{1:T}\right)=\frac{p\left(z_{1:T},x_{1:T}|\theta \right)p\left(\theta \right)}{p\left(z_{1:T}\right)}, \end{array}$$

where *p*(*z*_1:*T*_, *x*_1:*T*_|θ) is the likelihood of observed data and latent states given the model parameters θ, *p*(θ) is the prior distribution of θ, and *p*(*z*_1:*T*_) is the marginal likelihood or evidence. Because the posterior density is computationally intractable, an alternative approach is to use the MCMC method to generate samples from the joint posterior distribution of θ and *x*_1:*T*_ (21). These samples can then be used to estimate summary statistics such as posterior mean or credible intervals. Initially, given the current value of θ and *x*_1:*T*_, new values for θ^*^ and $x_{1:T}^{*}$ are sampled based on the density of the proposal $q\left(\theta^{*},x_{1:T}^{*}|\theta ,x_{1:T}\right)$. Then, θ and *x*_1:*T*_ will be updated by the new values θ^*^ and $x_{1:T}^{*}$ with probability:


(3) $$\begin{array}{l} \min\left\{\frac{p\left(\theta^{*},x_{1:T}^{*}|z_{1:T}\right)}{p\left(\theta ,x_{1:T}|z_{1:T}\right)}\frac{q\left(\theta ,x_{1:T}|\theta^{*},x_{1:T}^{*}\right)}{q\left(\theta^{*},x_{1:T}^{*}|\theta ,x_{1:T}\right)},1\right\}, \end{array}$$

where the posterior probability $p\left(\theta^{*},x_{1:T}^{*}|z_{1:T}\right)$ is evaluated by computing *p*(*z*_1:*T*_, *x*_1:*T*_|θ)*p*(θ) in Equation (2).

Based on the MCMC method, a sequence of samples will be generated to simulate the underlying epidemic dynamics, with the proposal density $q\left(\theta^{*},x_{1:T}^{*}|\theta ,x_{1:T}\right)$ determining the next state of the Markov chain and affecting the efficiency and accuracy of the method; however, finding an efficient proposal density for MCMC involves a balance between proposing accepted moves and exploring the distribution, which is challenging for the non-linear epidemic models we introduced in this study (22). Moreover, it is also difficult or impossible to evaluate the likelihood *p*(*z*_1:*T*_, *x*_1:*T*_|θ) in Equation (2) when the exact infection times are unknown (23). In this study, we first generate a new value for θ from the proposal density *q*(θ^*^|θ) and then sample $x_{1:T}^{*}$ independently from $p\left(x_{1:T}|\theta^{*},z_{1:T}\right)$ using a particle filtering algorithm. In this case, the proposal density can be defined as follows:


$$\begin{array}{l} q\left(\theta^{*},x_{1:T}^{*}|\theta ,x_{1:T}\right)=q\left(\theta^{*}|\theta \right)p\left(x_{1:T}^{*}|\theta^{*},z_{1:T}\right). \end{array}$$

The acceptance rate in Equation (3) can then be reformulated as follows:


$$\begin{array}{l} \;\frac{p\left(\theta^{*},x_{1:T}^{*}|z_{1:T}\right)}{p\left(\theta ,x_{1:T}|z_{1:T}\right)}\frac{q\left(\theta ,x_{1:T}|\theta^{*},x_{1:T}^{*}\right)}{q\left(\theta^{*},x_{1:T}^{*}|\theta ,x_{1:T}\right)}\\ =\frac{p\left(x_{1:T}^{*}|\theta^{*},z_{1:T}\right)p\left(\theta^{*}|z_{1:T}\right)}{p\left(x_{1:T}|\theta ,z_{1:T}\right)p\left(\theta |z_{1:T}\right)}\cdot \frac{q\left(\theta |\theta^{*}\right)p\left(x_{1:T}|\theta ,z_{1:T}\right)}{q\left(\theta^{*}|\theta \right)p\left(x_{1:T}^{*}|\theta^{*},z_{1:T}\right)}\\ =\frac{p\left(x_{1:T}^{*}|\theta^{*},z_{1:T}\right)p\left(z_{1:T}|\theta^{*}\right)p\left(\theta^{*}\right)/p\left(z_{1:T}\right)}{p\left(x_{1:T}|\theta ,z_{1:T}\right)p\left(z_{1:T}|\theta \right)p\left(\theta \right)/p\left(z_{1:T}\right)}\\ \;\cdot \frac{q\left(\theta |\theta^{*}\right)p\left(x_{1:T}|\theta ,z_{1:T}\right)}{q\left(\theta^{*}|\theta \right)p\left(x_{1:T}^{*}|\theta^{*},z_{1:T}\right)}\\ =\frac{p\left(z_{1:T}|\theta^{*}\right)p\left(\theta^{*}\right)q\left(\theta |\theta^{*}\right)}{p\left(z_{1:T}|\theta \right)p\left(\theta \right)q\left(\theta^{*}|\theta \right)}. \end{array}$$

Accordingly, the acceptance probability is given by:


(4) $$\begin{array}{l} \min\left\{\frac{p\left(z_{1:T}|\theta^{*}\right)p\left(\theta^{*}\right)q\left(\theta |\theta^{*}\right)}{p\left(z_{1:T}|\theta \right)p\left(\theta \right)q\left(\theta^{*}|\theta \right)},1\right\}, \end{array}$$

where the marginal likelihood $p\left(z_{1:T}|\theta^{*}\right)$ can be estimated by the following particle filtering algorithm.

### Particle filtering algorithm for marginal likelihood estimate

The PMCMC algorithm is an advanced version of the MCMC method that combines the benefits of particle filtering and MCMC techniques to create a more efficient proposal density (16, 18). This is achieved without the need for analytical computation of the likelihood *p*(*z*_1:*T*_, *x*_1:*T*_|θ) in Equation (2). In particular, the particle filtering algorithm employed in PMCMC allows for the numerical approximation of *p*(*x*_1:*T*_|θ, *z*_1:*T*_) by simulating unknown trajectories of state variables *x*_1:*T*_ from the process model. The key is to update particles sequentially over time so that at any time *t*, the weighted particles provide an approximation to the density *p*(*x*_1:*T*_|θ, *z*_1:*T*_). It allows for more efficient exploration of the posterior distribution, especially in high-dimensional and complex models, and can lead to faster convergence and improved accuracy of the estimates.

In the PMCMC algorithm, a number of *J* particles are chosen to simulate the trajectories of the state variables. The initial values of the model parameters in θ are first generated and arbitrarily assigned to these particles. For each particle *j*, we simulate the process model for *d* days (from time *t* = −*d*+1 to *t* = 0) and obtain the initial states of the variable $x_{1}^{j}$. Since our primary objective is to estimate epidemiological parameters based on the time series of reported cases, here the state variable *x*_1:*T*_ refers to the number of affected individuals during the time period from time *t* = 1 to *T*. After updating the state of the particles at time *t*, each particle is assigned a weight $w_{t}^{j}=p\left(z_{t}|x_{t}^{j},\theta \right)$, which is simply the probability of observing the data *z*_*t*_ given the state of the particle. The conditional marginal likelihood *p*(*z*_*t*_|*z*_1:*t*−1_, θ) can then be estimated as:


$$\begin{array}{l} \hat{p}\left(z_{t}|z_{1:t-1},\theta \right)=\frac{1}{J}\overset{J}{\underset{j=1}{\sum }}w_{t}^{j}. \end{array}$$

By the law of total probability, the marginal likelihood of the entire series of observations *z*_1:*T*_ given θ can be approximated as:


$$\begin{array}{l} \hat{p}\left(z_{1:T}|\theta \right)=\overset{T}{\underset{t=1}{\prod }}\hat{p}\left(z_{t}|z_{1:t-1},\theta \right)=\overset{T}{\underset{t=1}{\prod }}\left(\frac{1}{J}\overset{J}{\underset{j=1}{\sum }}w_{t}^{j}\right). \end{array}$$

In doing so, we can evaluate the acceptance probability in Equation 4 to perform MCMC sampling for θ^*^ and $x_{1:T}^{*}$.

After each time step *t*, particles are resampled using bootstrap filtering based on their normalized weight:


$$\begin{array}{l} {\bar{w}}_{t}^{j}=\frac{w_{t}^{j}}{\overset{J}{\underset{i=1}{\sum }}w_{t}^{i}}. \end{array}$$

Assume that the resampled parent index of the particle *j* is *k* at time *t*, then reset $x_{t-1}^{j}=x_{t-1}^{k}$ and propagate the particles by simulating the process model to the next observation time *t*. In doing so, the ancestry of the particles will be tracked so that a single trajectory (i.e., $x_{1:T}^{*}$) that represents the path of a single particle can be sampled. In summary, the particle filtering algorithm allows us to calculate the marginal likelihood $\hat{p}\left(z_{1:T}|\theta \right)$ and generate samples of $x_{1:T}^{*}$ from the state-space model. The algorithm must be executed for a minimum of *M* steps until all the parameters and the latent variables have converged. The pseudocode for the proposed PMCMC algorithm is described in Algorithm 1. The datasets and code for this study are available at the following GitHub link: https://github.com/Nanjing-Tech-University-CSIC/Bayesian-Inference-for-Emerging-Infectious-Diseases.

**Algorithm 1:**
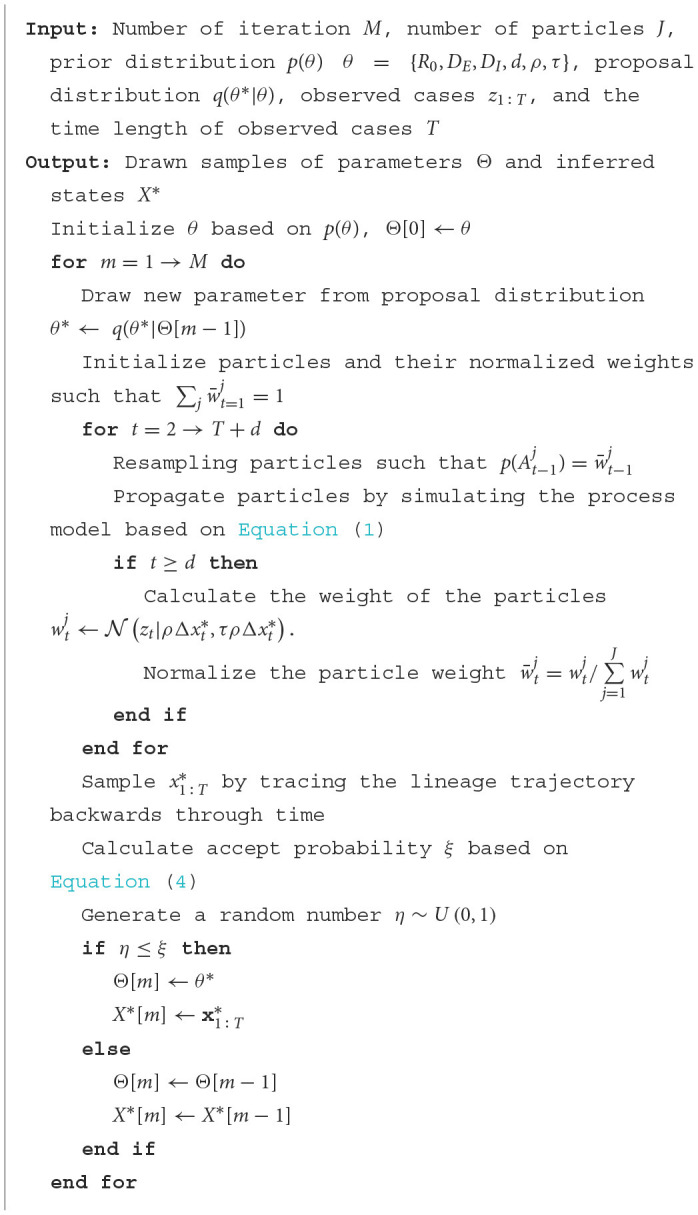
Pseudo-code for particle Markov chain Monte Carlo method.

## Synthetic experiments

In this section, we evaluate the performance of the proposed model and the PMCMC algorithm by conducting a set of synthetic experiments. We first generate time series of observation data *z*_1:*T*_ based on the proposed state-space model with predefined parameters θ. We then apply the PMCMC algorithm to these synthetic data to estimate the parameters θ. This process involves running the PMCMC algorithm on the generated time series *z*_1:*T*_, with the aim of recovering the predefined parameters. The effectiveness of our model and the PMCMC algorithm is assessed by comparing the estimated parameters with the original values of θ used in data generation. This comparison allows us to evaluate the precision of our approach in parameter estimation, as well as its robustness in handling synthetic datasets that mimic real-world epidemic scenarios. Through these experiments, our aim is to demonstrate the capability of our model and the PMCMC algorithm in reliably inferring key epidemiological parameters, an essential step toward validating their practical applicability in real-world epidemic analysis.

### Experimental settings

We initiate our study by simulating the stochastic SEIR model over time, using a set of predetermined parameters θ, including *R*_0_ = 2.2, *D*_*E*_ = 5.2, *D*_*I*_ = 2.7, ρ = 1, and τ = 0. In this context, setting ρ = 1 implies that every new infection is promptly identified. These initial parameters are selected based on information from existing studies, as mentioned by Li et al. (20). We also assume that the onset time of the epidemic occurs *d* = 10 days before the observation of the first infectious case. The simulation produces a series of data points that represent the progression of infections over time. To align the model more closely with real-world scenarios, where infection counts are integral values, we round the model output to the nearest integer. These rounded values, which form an integer-based time series, are subsequently fed into our observation model. Our analysis primarily centers on this integer time series, particularly examining the data post-emergence of initial infections.

With respect to the PMCMC algorithm, we assign the non-informative uniform prior distributions to the model parameters to be estimated in order to minimize the effects of the priors on the posterior results. Specifically, we set *R*_0_~*U*(0, 4), *D*_*E*_~*U*(0, 10), *D*_*I*_~*U*(0, 6) and *d*~*U*(0, 20). The initial value of each parameter in θ is generated randomly on the basis of its prior distribution. The proposal distribution for each parameter is set as follows: $q\left(R_{0}|R_{0}\right)=N\left(R_{0}|R_{0},0.2\right)$, $q\left(D_{E}|D_{E}\right)=N\left(D_{E}|D_{E},0.5\right)$, $q\left(D_{I}^{*}|D_{I}\right)=N\left(D_{I}|D_{I},0.5\right)$ and q(d∣d)=N(d∣d,0.5). We execute the PMCMC algorithm for 50,000 iterations, discarding the first 5,000 as burn-in. We then use the last 90% iterations to calculate the posteriors for each parameter and their 95% credible intervals.

### Experimental results

#### Inference accuracy of model parameters

**Figure 1:**
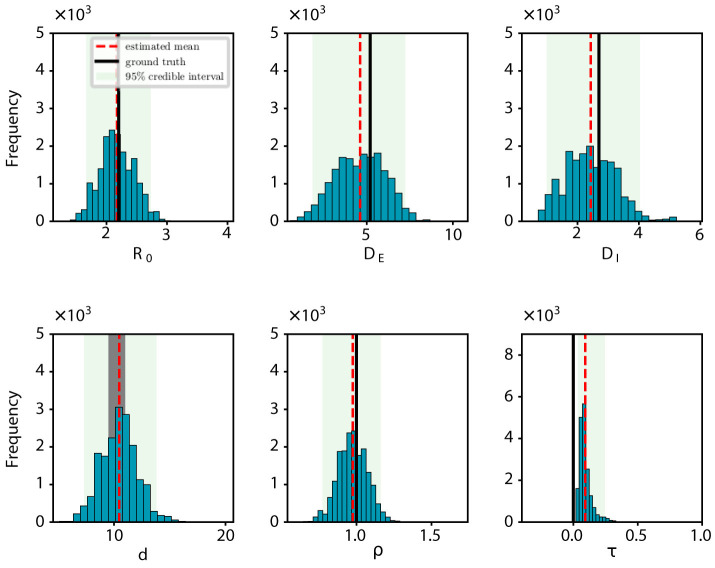
Posterior density estimates for unknown model parameters in synthetic experiments. The horizontal axis represents the range of parameter values, while the vertical axis represents the frequency of samples from the posterior distribution generated by the PMCMC algorithm. The mean estimates are shown as red dotted lines, the 95% CIs are represented by the green regions, and the true values are shown as black lines.

Initially, we evaluated the accuracy of our parameter inference method. Posterior density estimates for unknown model parameters θ are shown in Figure 1. The horizontal axis represents the range of parameter values, while the vertical axis represents the frequency of samples from the posterior distribution generated by the PMCMC algorithm. The mean estimate is shown as a red dotted line, the 95% credible interval (CI) is represented by the green region, and the true value is shown as a black line. Our predictive analysis yields the following mean estimates with the corresponding 95% credible intervals (CIs): *R*_0_ = 2.17 (95% CI: [1.66, 2.73]), *D*_*E*_ = 4.61 (95% CI: [1.85, 7.24]), *D*_*I*_ = 2.43 (95% CI: [1.00, 4.04]), *d* = 10.48 (95% CI: [7.30, 13.81]), ρ = 0.98 (95% CI: [0.77, 1.16]), τ = 0.09 (95% CI: [0.03, 0.25]). The estimated model parameters closely match their true values, and all estimates fall within their respective 95% CIs. Figure 4A displays the fitting outcomes of the proposed model and algorithm. The coefficient of determination (*R*^2^) is 0.98, suggesting a strong resemblance between the fitting curve and the actual data. These results highlight the accurate estimation of key epidemiological parameters from limited epidemic data using our model and algorithm, demonstrating their effectiveness in understanding early-stage epidemic dynamics.

#### Impact of the length of the observational time series

**Figure 2:**
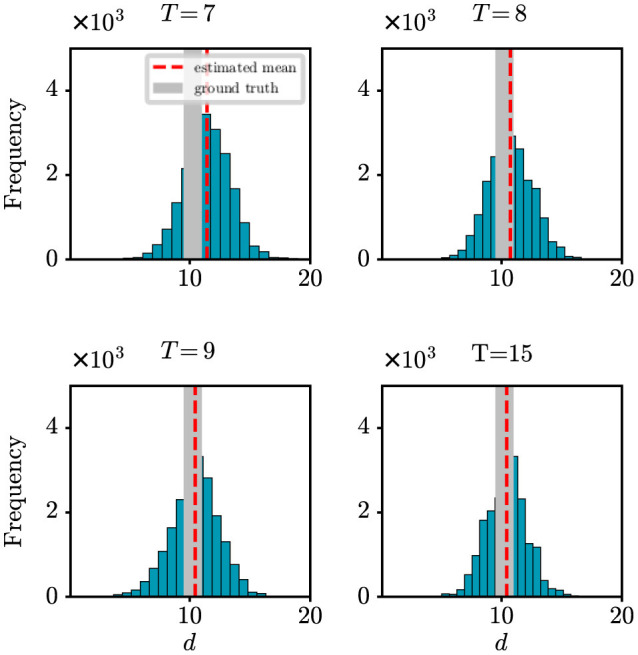
Posterior density estimate for the onset time based on observational time series of varying lengths *T*. The red dotted line indicates the mean estimate of outcomes, while the black region highlights the ground-truth value.

In the early stages of an epidemic, accurately determining epidemiological parameters from brief time series is key to quickly developing effective preventive measures. Therefore, it is vital to assess how the length of the observational time series affects the inference results. In our synthetic tests, we explored how various lengths of observational time series impact the precision of determining an epidemic's onset time. Figure 2 shows the posterior density estimates of the onset time *d* for different lengths of the observational time series *T*= 7, 8, 9, and 15. The red dotted lines indicate the mean estimates of outcomes, while the black regions highlight the actual values of the ground truth. We observed that longer time series generally yield more precise predictions. In particular, with just 8 days of data, the prediction accuracy is already commendably high, suggesting that our approach can deliver reliable results even with relatively short time series.

#### Robustness of the proposed model and algorithm

**Figure 3:**
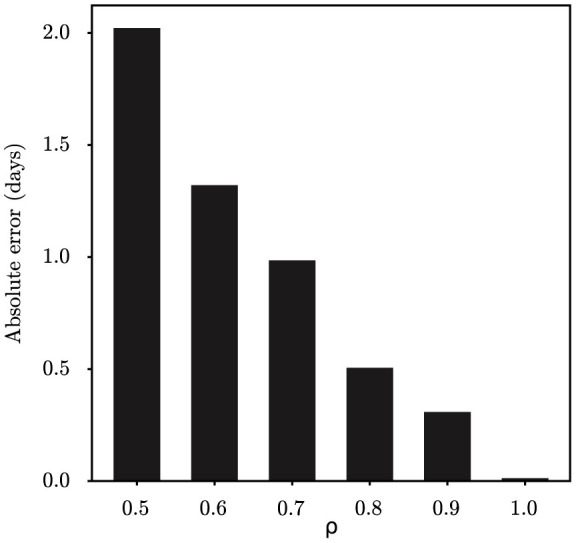
The estimation error of the onset time *d* under different settings of detection probability ρ.

Due to various factors, such as incomplete surveillance systems, there is a tendency for observed data to be underestimated in the early stages of an epidemic. In such scenarios, it becomes particularly crucial to evaluate the robustness of our model and algorithm. In this paper, we introduced the parameter ρ in our observation model to represent the probability of detecting newly infected cases. Here, we aim to assess how changes in detection probability ρ affect our model's ability to accurately determine the onset time of an epidemic. Figure 3 shows the absolute error (days) with respect to the onset time *d* under different settings of ρ. It can be found that as the value of ρ decreases, the difference between the estimation error also decreases. However, it is important to note that when the detection rate reaches around 70%, our method can achieve satisfactory prediction results. It demonstrates how our model maintains its effectiveness and accuracy in predicting the onset time of an epidemic, even under varying detection probabilities, reinforcing the reliability of our findings in practical scenarios. This result offers valuable support for the credibility of the outcomes obtained from our real-world case studies in the following section.

**Figure 4:**
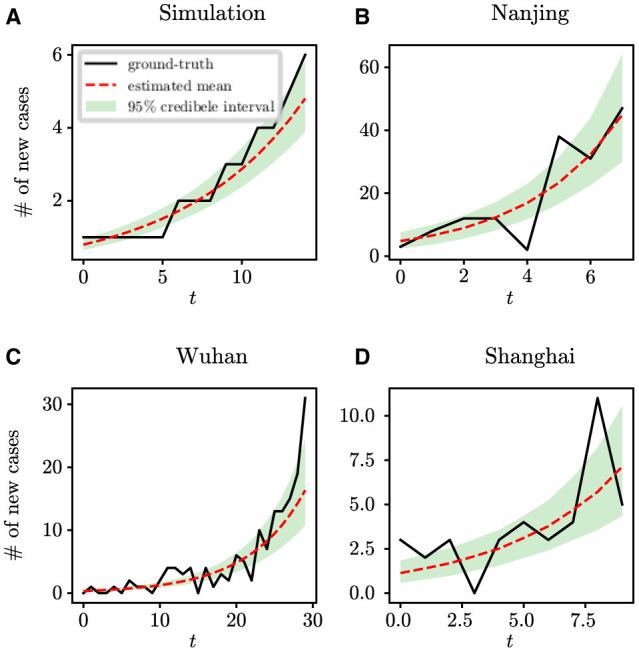
The fitting results of the proposed model and algorithm with respect to the ground-true time series. **(A)** Synthetic data, **(B)** COVID-19 data in Nanjing, **(C)** COVID-19 data in Wuhan, and **(D)** COVID-19 data in Shanghai.

## Real-world case studies

In this section, we use the state-space model and the PMCMC algorithm to conduct retrospective studies on COVID-19 outbreaks in Wuhan, Shanghai and Nanjing, China. The objective is to employ Bayesian inference techniques to accurately model and understand the spread of infectious diseases in its early stages, thus improving our ability to predict, manage, and mitigate future outbreaks effectively.

### The COVID-19 outbreak in Wuhan, China

The COVID-19 outbreak in Wuhan, China, in December 2019, marked a pivotal moment in the global progression of the disease. The virus, later identified as SARS-CoV-2, quickly overwhelmed Wuhan's healthcare infrastructure, signaling a major public health emergency. Characterized by efficient human-to-human transmission, the virus led to an exponential increase in cases. Despite the government having promptly implemented unprecedented control measures, including a thorough lockdown and extensive travel restrictions, these efforts failed to prevent further spread of the epidemic, ultimately resulting in incalculable losses. The Wuhan outbreak underscored the urgent need for global awareness and response strategies, which led to international efforts to understand the epidemiological and biological characteristics of the virus. Our objective is to conduct retrospective analyzes and draw on lessons from previous outbreaks, thus fortifying our ability to predict, control, and reduce the impact of future infectious threats.

**Figure 5:**
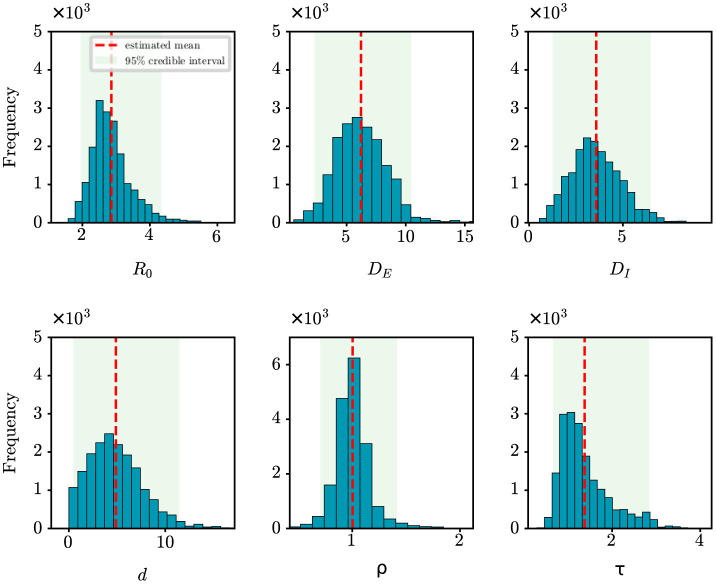
Posterior density estimates of unknown model parameters for the COVID-19 outbreak in Wuhan, China. The horizontal axis represents the range of parameter values, while the vertical axis represents the frequency of samples from the posterior distribution generated by the PMCMC algorithm. The mean estimates are shown as red dotted lines, the 95% CIs are represented by the green regions.

We perform analysis on the initial 124 confirmed cases in Wuhan, documented by Li et al. (20), which spanned 30 days from December 8th, 2019 to January 6th, 2020. The population size of Wuhan is set at 11,000,000. Figure 5 shows the estimates of unknown epidemiological parameters for the COVID-19 outbreak in Wuhan, China. The mean estimations and their 95% CIs for the parameters are: *R*_0_ = 2.86 (95% CI: [1.95, 4.34]), *D*_*E*_ = 6.22 (95% CI: [2.28, 10.46]), *D*_*I*_ = 3.58 (95% CI: [1.25, 6.49]), *d* = 4.87 (95% CI: [0.46, 11.47]), ρ = 1.00 (95% CI: [0.70, 1.41]), τ = 1.38 (95% CI: [0.67, 2.84]). There is no doubt that it is quite challenging to validate the accuracy of these findings using epidemiological or empirical methods. However, it should be noted that some of our results exhibit a remarkable alignment with those reported in existing research (20, 24, 25). For example, Li et al. determined that the value of *R*_0_ was 2.2 (95% CI: [1.4, 3.9]) and reported that the mean incubation period was 5.2 days (95% CI: [4.1, 7.0]) (20); Pan et al. calculated that the effective reproduction number fluctuated around 3.0 before January 26, 2020 (24); Read e al. estimate a basic reproductive number of 3.11 (95% CI: [2.39, 4.13]) using a transmission model (25). All these findings corroborate the inference results drawn in this study. In addition, the fitting results depicted in Figure 4C demonstrate that the fitting curve closely approximates the actual observation data, with the coefficient of determination *R*^2^ of 0.86.

### The COVID-19 epidemic in Shanghai, China

In March 2022, Shanghai experienced a significant wave of COVID-19 cases, which presented new challenges to the public health system of the city. The surge in infections was attributed to the emergence of a highly transmissible Omicron variant of the virus. This wave of the epidemic in Shanghai can be traced back to March 1st, 2022. On that day, during a press conference on COVID-19 prevention and control in Shanghai, a locally transmitted case was reported ([https://china.huanqiu.com/article/470y1untEKO]). The community cultural activity center where the case was identified was classified as a medium-risk area. As the trace of the epidemic progressed, it was determined that the outbreak originated in the Xuhui district. This area served as a centralized quarantine facility for incoming travelers, where abnormal results were detected during routine nucleic acid tests for staff members. The viral strain responsible for this outbreak is highly contagious and extremely covert, leading to a significant number of asymptomatic carriers. Ultimately, this resulted in the explosive spread of the epidemic.

**Figure 6:**
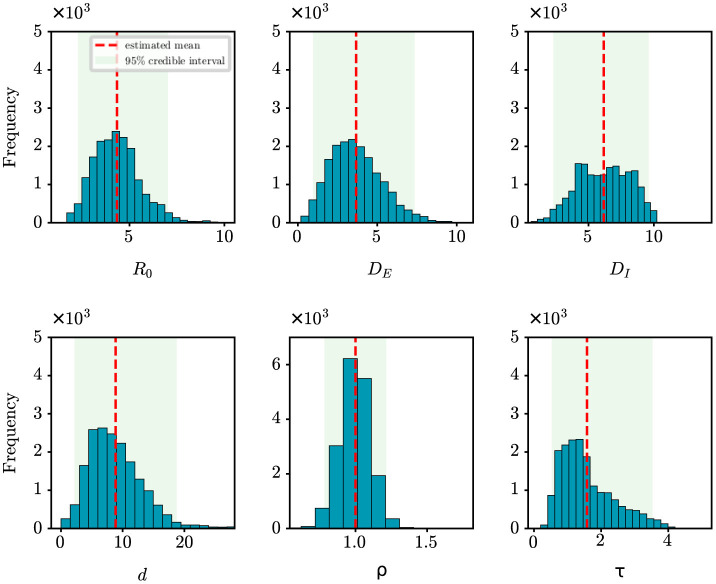
Posterior density estimates of unknown model parameters for the COVID-19 epidemic in Shanghai, China. The horizontal axis represents the range of parameter values, while the vertical axis represents the frequency of samples from the posterior distribution generated by the PMCMC algorith. The mean estimates are shown as red dotted lines, the 95% CIs are represented by the green regions.

For the retrospective analysis of the epidemic in Shanghai, we gather the incidence data of the first 10 days as reported by the NHC (http://www.nhc.gov.cn/xcs/xxgzbd/gzbd_index.shtml). The data covers the period from March 1st, 2022 to March 10th, 2022. The population size is established at 24,900,000. Figure 6 shows the posterior density estimates of unknown epidemiological parameters. The mean estimations and 95% CIs for the parameters are estimated as: *R*_0_ = 4.34 (95% CI: [2.28, 7.03]), *D*_*E*_ = 3.67 (95% CI: [0.96, 7.36]), *D*_*I*_ = 6.17 (95% CI: [2.31, 9.61]), *d* = 8.85 (95% CI: [2.17, 18.77]), ρ = 1.00 (95% CI: [0.78, 1.21]), τ = 1.58 (95% CI: [0.53, 3.53]). Similar to the case study of the COVID-19 epidemic in Wuhan, we find that our results are consistent with the epidemiological characteristics of the Omicron variant, which is evident and supported by the literature (26, 27). For example, Cai et al. estimated that *R*_0_ of the Omicron BA.2 variant was 3.9 at the beginning of the 2022 outbreak in Shanghai (26); Wu et al. determined that the mean incubation period of COVID-19 was 3.42 days (95% CI: [2.88, 3.96]) for the Omicron variant through meta-analysis (27). Furthermore, Figure 4D shows how well our model and algorithm fit compared to actual observations. It can be found that the coefficient of determination *R*^2^ is 0.62, which is much lower than the result of our synthetic experiment (*R*^2^ = 0.98). The rationale behind this is that the inference of model parameters is based on only nine days of observation data, during which the data exhibited significant fluctuations in the initial phase of the epidemic.

### The COVID-19 epidemic in Nanjing, China

In July 2021, Nanjing experienced a COVID-19 outbreak primarily attributed to the introduction of the virus via an international flight. The origin of the outbreak was first identified at Nanjing Lukou International Airport. It was reported that cleaners who had worked on a flight that arrived on July 10th, 2021 from Russia were infected with the virus. Despite stringent control measures in place, the virus quickly spread through the airport, affecting both staff and passengers. This incident led to subsequent local transmissions in Nanjing and spread to several other cities in China, causing a significant increase in COVID-19 cases.

**Figure 7:**
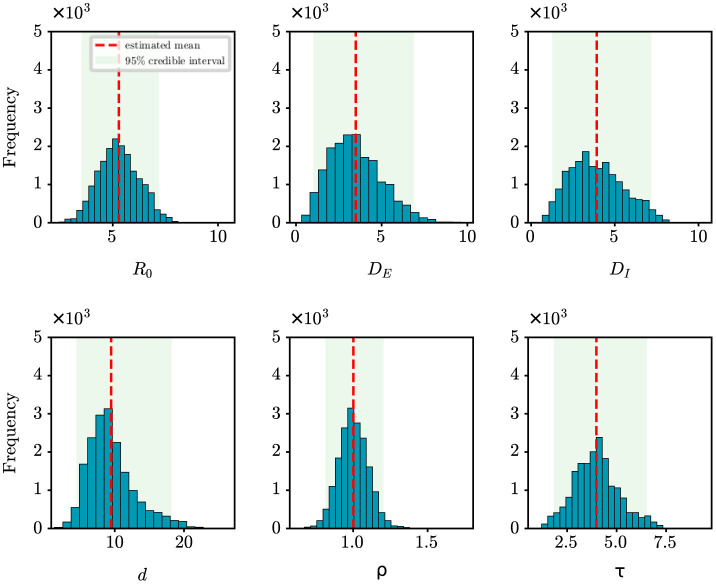
Posterior density estimates of unknown model parameters for the COVID-19 outbreak in Nanjing, China. The horizontal axis represents the range of parameter values, while the vertical axis represents the frequency of samples from the posterior distribution generated by the PMCMC algorith. The mean estimates are shown as red dotted lines, the 95% CIs are represented by the green regions.

For retrospective analysis, we collect the incidence data for the initial 8 days from JSCH (http://wjw.jiangsu.gov.cn). The data span from July 20th, 2021 to July 27th, 2021. The population size of Nanjing is set at 9,650,000. Figure 7 shows the estimation results of unknown epidemiological parameters. The mean estimations and 95% CIs for the parameters are estimated as *R*_0_ = 5.29 (95% CI: [3.52, 7.19]), *D*_*E*_ = 3.49 (95% CI: [1.01, 6.89]), *D*_*I*_ = 3.92 (95% CI: [1.26, 7.19]), *d* = 9.48 (95% CI: [4.46, 18.22]), ρ = 1.00 (95% CI: [0.81, 1.20]), τ = 3.98 (95% CI: [1.84, 6.53]). The findings of our study align well with the epidemiological characteristics of the Delta variant, as reported in previous studies (27–29). For example, Wu et al. have shown through a meta-analysis that the mean incubation period of COVID-19 was 4.41 days (95% CI: [3.76, 5.05]) for the Delta variant (27); Zhang et al. have shown that the mean incubation period is 4.4 days [95% CI: [3.9, 5.0] for the Delta variant epidemic in Guangzhou, China (29). Compared to the results mentioned above, the estimated value of *D*_*E*_ for Nanjing is slightly shorter. The reason is that the Nanjing outbreak originated at the airport, where stricter control measures were in place, allowing potential cases to be quickly identified by nucleic acid testing. Meanwhile, the effectiveness of our approach in capturing the dynamics of the outbreak is visually depicted in Figure 4B, with an *R*^2^ value of 0.78. Furthermore, our findings also indicate that the onset of the epidemic occurred 9.48 days prior to the first reported case, pinpointing the date to July 10th, 2021. Epidemiological investigations have revealed that this outbreak in Nanjing was indeed triggered by a flight on this specific date. This observation further validates the efficacy of our algorithm in accurately determining critical epidemiological characteristics.

## Discussion

In the face of the ongoing emergence of infectious diseases that cause considerable economic and health damage, it is essential to accurately and quickly identify the date of onset of patient zero and other key epidemiological parameters. Accurate estimation is crucial for quickly isolating individuals involved in the outbreak, curtailing the further spread of the disease (30), forming timely policies, and effectively mitigating the adverse impacts of the epidemic (31). In this study, we develop a Bayesian inference method to provide an accurate estimate of key epidemiological parameters of an epidemic, including the basic reproduction number *R*_0_, the latent period *D*_*E*_, and the infectious period *D*_*I*_, even with noisy and limited observation data. Specifically, a state-space model is proposed to simulate the stochastic and non-linear dynamics of epidemics, while the PMCMC algorithm is used to infer the underlying epidemiological parameters.

Through synthetic experiments, we have validated the efficacy of the proposed state-space model and the PMCMC algorithm in accurately estimating model parameters. For example, when estimating the onset time (*d*) against the actual value of *d* = 10 with different lengths of observational time series (*T*), we have obtained the following results: *d* = 11.43 for *T* = 7, *d* = 10.74 for *T* = 8, *d* = 10.45 for *T* = 9, and *d* = 10.44 for *T* = 15. In particular, with an 8-day observational series, the estimation error for the onset time is < 1 day. Moreover, we have also evaluated the robustness of our model and algorithm by examining the estimated onset time in different settings of observation probability ρ. We have found that even when the probability reaches around 70%, the estimation error is < 1 day. All these experimental results validate the effectiveness of our approach in understanding the epidemiological characteristics of an infectious disease in its early stages of transmission, demonstrating its potential utility in investigating and managing future outbreaks.

The complexity of epidemic dynamics in the real world far exceeds that of synthetic studies. Conducting retrospective analyses on COVID-19 outbreaks in Wuhan, Shanghai and Nanjing, China, offers deeper insights into actual epidemic patterns. For these case studies, we have used incidence data that span varying lengths to infer key epidemiological parameters. Although the stochastic SEIR model is sensitive to the initial values of parameters, our study begins by assigning non-informative uniform priors to these parameters. Through Bayesian inference, we generally calculate the posterior distributions of the parameters, regardless of the initial configurations of the model parameters. We have observed that the more days of epidemic data we collected, the better our method fits to the real data, as indicated by higher values of *R*^2^. For example, in analyzing the Wuhan outbreak with 30 days of data, we achieved an *R*^2^ of 0.86, significantly higher than that of Shanghai (*R*^2^ = 0.62) and Nanjing (*R*^2^ = 0.78), which are based only on the first 10 and 8 days of outbreak data, respectively. In addition, the quality of the data significantly influences the inference outcomes, especially in the early stages of an epidemic. As shown in Figure 4, the quality of the data for Shanghai and Nanjing is not ideal, leading to comparatively lower fitting results. This underscores the critical role that data quality plays in accurately modeling and understanding epidemic trends. To increase the *R*^2^ value, one feasible approach is to improve data quality by strengthening the disease surveillance system. Another is to collect more data to better characterize the transmission dynamics.

Even with limited early-stage data, retrospective analysis using the proposed model and algorithm yields results of practical significance. For example, our analysis of early Wuhan outbreak data deduced a basic reproduction number *R*_0_ = 2.86 (95% CI: [1.95, 4.34]), which is consistent with existing research findings (20, 24, 25). Furthermore, our analysis of the initial data from the Shanghai outbreak led to the deduction of epidemiological parameters that are consistent with the characteristics of the Omicron variant (26, 27). Regarding the onset time of the Wuhan and Shanghai outbreaks, there is no widely accepted evidence to verify our inferred results. However, in the case of the Nanjing epidemic, we have managed to determine the date of patient zero's initial presence as July 10th, 2021, based on the data collected during the first 8 days of the outbreak. Our analytical results are fully consistent with the findings of epidemiological investigation, which demonstrates the applicability and relevance of our approach in understanding the dynamics of epidemics in the real world.

It is important to note that the epidemiological parameters can vary between different cities and time periods. One contributing factor is the diversity of disease intervention policies as the epidemic progresses in each city. In addition, the virus itself evolves and exhibits different characteristics during different transmission periods (32). For example, the Delta variant was prevalent in Nanjing (33), while Shanghai experienced the spread of the Omicron strain (34). Despite analyzing identical strains of SARS-CoV-2, there may be minor variations between our estimates and those found in reference studies, which can be ascribed to the constrained dataset employed in our analysis. However, by employing the Bayesian inference approach, the PMCMC algorithm is able to provide posterior densities for epidemiological parameters, along with 95% CIs. Despite minor discrepancies, all the estimated values of the reference studies fall within the range of these 95% CIs, indicating the robustness of our approach.

In summary, the proposed method has shown potential in uncovering early-stage epidemiological parameters and estimating the initial emergence of patient zero. However, there are notable limitations in our current approach. First, we assume that there is no delay in reporting cases and that epidemiological parameters remain constant over time. Although it is plausible to assume stable epidemiological parameters during the early stages of an epidemic, delayed reporting of confirmed cases could lead to varied inference outcomes. To address this issue, improvements in the monitoring system might be required. Second, our model does not account for the impact of asymptomatic COVID-19 infections, which blur the lines between exposed and infected states. Introducing a compartment for asymptomatic cases could improve the precision of our modeling. Third, while we used the SEIR model, it does not fully account for critical factors such as demographic variations, population movement, and isolation measures, all of which are vital in epidemic dynamics. A more holistic model that includes these elements would improve our understanding of epidemics. Finally, the predictive accuracy of our method might decrease with shorter data series or inaccurate data. Therefore, future research should focus on improving the assessment of transmission risks using limited and possibly imperfect data, as well as improving predictions by incorporating additional knowledge and data sources.

## Conclusion

In this study, we have proposed a Bayesian inference approach to explore the essential epidemiological parameters of emerging infectious diseases. We began by simulating the stochastic and non-linear dynamics of an epidemic using state-space modeling. We then introduced the particle Markov chain Monte Carlo (PMCMC) method to infer the underlying epidemiological parameters, including the basic reproduction number, the latent period, the infectious period, and the onset time (marked by the appearance of the patient zero). Having validated the effectiveness of the proposed model and algorithm through synthetic experiments, we subsequently applied them to real-world case studies, such as the COVID-19 outbreaks in Wuhan, Shanghai and Nanjing, China. The results have indicated that the proposed model and algorithm can accurately estimate key model parameters and successfully predict the future trend of the outbreak. In conclusion, our approach has shown the potential to be valuable in improving our understanding of the transmission dynamics of emerging epidemics, thus facilitating better responses to health crises and mitigating their adverse impacts.

## Data availability statement

Publicly available datasets were analyzed in this study. This data can be found here: http://www.nhc.gov.cn/xcs/xxgzbd/gzbd_index.shtml; http://wjw.jiangsu.gov.cn/; https://www.nejm.org/doi/full/10.1056/nejmoa2001316.

## Author contributions

BS: Conceptualization, Data curation, Formal analysis, Funding acquisition, Methodology, Project administration, Supervision, Validation, Writing—original draft, Writing—review & editing. SY: Methodology, Validation, Writing—original draft, Investigation, Visualization. QT: Formal analysis, Investigation, Methodology, Validation, Writing—review & editing. LZ: Formal analysis, Investigation, Validation, Writing—review & editing. YL: Conceptualization, Formal analysis, Methodology, Validation, Visualization, Writing—review & editing. XZ: Data curation, Formal analysis, Investigation, Validation, Writing— review & editing. JL: Conceptualization, Formal analysis, Funding acquisition, Methodology, Project administration, Supervision, Writing—review & editing.
